# Antimicrobial activities of green synthesized gums-stabilized nanoparticles loaded with flavonoids

**DOI:** 10.1038/s41598-019-39528-0

**Published:** 2019-02-28

**Authors:** Ayaz Anwar, Abdulkader Masri, Komal Rao, Kavitha Rajendran, Naveed Ahmed Khan, Muhammad Raza Shah, Ruqaiyyah Siddiqui

**Affiliations:** 1 Department of Biological Sciences, School of Science and Technology, Sunway University, Subang Jaya, 47500 Selangor Malaysia; 2 H.E.J. Research Institute of Chemistry, International Center for Chemical and Biological Sciences, University of Karachi, Karachi, 75270 Pakistan

## Abstract

Herein, we report green synthesized nanoparticles based on stabilization by plant gums, loaded with citrus fruits flavonoids Hesperidin (HDN) and Naringin (NRG) as novel antimicrobial agents against brain-eating amoebae and multi-drug resistant bacteria. Nanoparticles were thoroughly characterized by using zetasizer, zeta potential, atomic force microscopy, ultravoilet-visible and Fourier transform-infrared spectroscopic techniques. The size of these spherical nanoparticles was found to be in the range of 100–225 nm. The antiamoebic effects of these green synthesized Silver and Gold nanoparticles loaded with HDN and NRG were tested against *Acanthamoeba castellanii* and *Naegleria fowleri*, while antibacterial effects were evaluated against methicillin-resistant *Staphylococcus aureus* (MRSA) and neuropathogenic *Escherichia coli* K1. Amoebicidal assays revealed that HDN loaded Silver nanoparticles stabilized by gum acacia (GA-AgNPs-HDN) quantitatively abolished amoeba viability by 100%, while NRG loaded Gold nanoparticles stabilized by gum tragacanth (GT-AuNPs-NRG) significantly reduced the viability of *A. castellanii* and *N. fowleri* at 50 µg per mL. Furthermore, these nanoparticles inhibited the encystation and excystation by more than 85%, as well as GA-AgNPs-HDN only completely obliterated amoeba-mediated host cells cytopathogenicity. Whereas, GA-AgNPs-HDN exhibited significant bactericidal effects against MRSA and *E. coli* K1 and reduced bacterial-mediated host cells cytotoxicity. Notably, when tested against human cells, these nanoparticles showed minimal (23%) cytotoxicity at even higher concentration of 100 µg per mL as compared to 50 µg per mL used for antimicrobial assays. Hence, these novel nanoparticles formulations hold potential as therapeutic agents against infections caused by brain-eating amoebae, as well as multi-drug resistant bacteria, and recommend a step forward in drug development.

## Introduction

Brain-eating amoebae are pathogenic protists and causative agents for deadly central nervous system (CNS) infections including primary amoebic encephalitis (PAM) and granulomatous amoebic encephalitis (GAE)^1^. Infections caused by brain-eating amoebae are rare but fatal and are of global concern due to increased exposure of public water-related activities combined with global warming^2^. Due to their rarity and/or lack of awareness, these diseases are challenging to diagnose and difficult to treat because of their ability to form resistant cysts^3^. Currently, a mixture of drugs including Amphotericin B, Chlorhexidine, Voriconazole, Miltefosine, Pentamidine and others is used in the management of infections caused by brain-eating amoebae, but the prognosis remains extremely poor and the mortality rate remains more than 90%^4,5^. Multi-drug resistant (MDR) bacteria such as methicillin-resistant *Staphylococcus aureus* (MRSA) and *E. coli* are more common causes of infectious diseases including urinary tract infections (UTI), meningitis, gastroenteritis, respiratory and skin diseases, etc^6,7^.

HDN is classified as bio-flavonoid, chemically belongs to parent compound flavones (a class of flavonoid) and contains disaccharide rutinose^8^. HDN is a leading compound of citrus fruits, isolated mainly from rinds of some citrus species e.g., bitter orange, sweet orange, and satsuma mandarin^9^. HDN possesses strong antiinflammatory, antiarthritic, antioxidant, anti-carcinogenic, antidiabetic and antihypertensive properties^10,11^. NRG is another flavonoid extracted from citrus fruits known for its versatile pharmacological values. It has been widely studied due to its antioxidant, anti-cancer and anti-inflammatory potentials^12,13^. However, the true therapeutic potency of flavonoids is generally retarded by their pH intolerance, easy oxidation, poor solubility etc.^14^. The shortcoming of lower water solubility of NRG has presented challenges in the drug development which leads to its poorer therapeutic efficacy^15,16^. Green chemistry is commonly utilized to synthesize such type of green nanoparticles for drug delivery purpose with biological safe and nontoxic materials^17^. For instance, natural materials which include plant extracts and by products from different plants have been used to synthesize green nanoparticles. Gum tragacanth (GT) is a complex polysaccharide obtained from the extract of genus *Astragalus*. It consists of a mixture of heterogeneous and branched hydrophilic polysaccharides with molecular weight in the range of 840 kDa. Hydrolysis of GT produces variety of sugars including D-galactose, D-xylose, L-fucose, L-arabinose, D-galacturonic acid, L-rhamnose etc. Gums from different plants are highly biocompatible, easily available and cost effective. Gum acacia (GA) is a biocompatible polymer obtained from trees. GA is an anionic branched polysaccharide which is widely employed in food and pharmaceutics as thickener, stabilizer, emulsifier, and it is also being used in drug delivery applications as a controlled release agent^18,19^. Hence, nontoxic and biocompatible properties of easily available gums made them useful for drug delivery system therefore gums stabilized/reduced nanoparticles can be an innovative nano-carrier for flavonoids delivery.

Nanoparticles based materials have recently shown tremendous *in vitro* potential against brain-eating amoebae. For example, Lemke *et al*., reported nanosuspension delivery system for Amphotericin B to brain against *Balamuthia mandrillaris* infection^20^. Previously, our research showed that Amphotericin B, Nystatin and Fluconazole conjugated AgNPs showed enhanced antiamoebic activity against brain-eating *N. fowleri* and *A. castellanii*^21,22^. Recently, natural compounds such as cinnamic acid^23^, periglaucine A, betulinic acid^24^, and tannic acid^25^ coated nanomaterials have been shown to be effective against *A. castellanii*. Based on these reports, we synthesized HDN and NRG loaded silver and gold nanoparticles stabilized by plant gums GA and GT respectively by utilizing green synthesis protocols. The design and synthesis of these materials is unique, robust and eco-friendly. The nanomaterials are thoroughly characterized by using various spectroscopic and microscopic techniques. These green-synthesized nanomaterials are being reported for the first time against brain-eating amoebae *A. castellanii* and *N. fowleri*, and against multi-drug resistant bacteria MRSA and neuropathogenic *E. coli* K1. HDN loaded GA stabilized nanoparticles showed significant amoebicidal and bactericidal efficacy, while minimal cytotoxicity against human cells. These nanoparticles hold promise for further evaluation of mechanism and *in vivo* studies against infectious diseases caused by free-living amoeba and multi-drug resistant bacteria.

## Method and Materials

### Materials

GA and GT were obtained from local market. HDN, NRG and Silver nitrate (AgNO_3_) purchased from Sigma-Aldrich Germany. Tetrachloroauric (III) acid trihydrate (HAuCl4-3H2O) was purchased from Merck. Deionized water was used for all formulations.

### Preparation of gum solutions

Stock solutions of GA and GT were prepared by dissolving respective gums in deionized water at 4 mg/mL and 8 mg/mL concentration respectively. The solutions were stirred for 24 h at room temperature to ensure complete dissolution of gums. The gum solutions were filtered to separate the undissolved materials if any. The prepared gum solutions were used to synthesize the gum stabilized nanoparticle.

### Green synthesis of GA-AgNPs and GT-AuNPs

For GA-AgNPs, silver nitrate solution (9 mg/ml) was added in equal volume (1:1 v/v) to the gum solution (3 mg/ml) which were stirred magnetically at 200 × g for 2 hr at 60 °C. GA-AgNPs formation was indicted by color change from colorless to pale yellow then converted to light brown. For GT-AuNPs, 0.1 mL of 5 mM gold aqueous solution was added to GT solution (1 mL, 5 mg/mL) and the reaction mixture was magnetically stirred at 200 × g for 4 h at 60 °C. The color change from colorless to deep purple indicated the reduction of gold and formation of GT-AuNPs. The formation of GA-AgNPs and GT-AuNPs was confirmed by determination of surface plasmon resonance using UV visible spectrophotometer (UV-240, Shimadzu).

### HDN and NRG loading on gum stabilized nanoparticles

HDN and NRG loaded gum stabilized nanoparticles were synthesized as described previously^17,26^. Briefly, 0.5 mg/mL HDN was added to above GA-AgNPs solution, and the reaction mixture was magnetically stirred at 200 × g at room temperature for 24 h. HDN loaded GA-AgNPs-HD were obtained in the form of pellet upon centrifugation at 12,000 × g for 30 min. To load NRG on GT-AuNPs, nanoparticles were centrifuged at 10,000 × g for 25 min and the collected nanoparticles pellet was re-dispersed in deionized water with the addition of 0.5 mg/mL final concentration of NRG in GT-AuNPs. The mixture was left for stirring at room temperature at 200 × g for 24 h.

### Characterization of nanoparticles size, polydispersity index (PDI), zeta potential and surface morphology

The size and PDI of nanoparticles were investigated through zetasizer (Malvern instrument UK). Nanoparticles were transferred into the plastic cuvette and read by using water as dispersant at 25 °C. Zeta potential was measured by using dip cell which was dipped into the cuvette containing diluted sample at 25 °C and read on the instrument. Morphology of the nanoparticles was determined through AFM (AFM, Agilent 5500). Colloidal samples were loaded on to the mica slide and left for air drying avoiding contamination from environment. Slides mounted on the microscope were visualized after drying, under non-contact mode.

### FT-IR study

FT-IR analysis of nanoparticles in comparison to gums and flavonoids alone were carried out by diluting the samples with KBr to form a pallet. The spectra were recorded in the range of 400–4000 cm^−1^ using FT-IR spectrometer (Shimadzu Kyoto, Japan).

### Determination of flavonoids loading efficiency

The loading of HDN/NRG on nanoparticles was determined spectrophotometrically. First the nanoparticles were centrifuged at 12,000 × g for 30 min. Supernatant (containing free drug) was discarded and pellet was collected and re-dispersed in methanol up to the final volume. HDN and NRG were detected and quantified at 285 nm^17,26^. The percentage of encapsulated flavonoids were calculated by using this formula:

$$ \% {\rm{F}}{\rm{l}}{\rm{a}}{\rm{v}}{\rm{o}}{\rm{n}}{\rm{o}}{\rm{i}}{\rm{d}}\,{\rm{l}}{\rm{o}}{\rm{a}}{\rm{d}}{\rm{e}}{\rm{d}}\,=\,({\rm{A}}{\rm{m}}{\rm{o}}{\rm{u}}{\rm{n}}{\rm{t}}\,{\rm{o}}{\rm{f}}\,{\rm{f}}{\rm{l}}{\rm{a}}{\rm{v}}{\rm{o}}{\rm{n}}{\rm{o}}{\rm{i}}{\rm{d}}\,{\rm{l}}{\rm{o}}{\rm{a}}{\rm{d}}{\rm{e}}{\rm{d}}/{\rm{T}}{\rm{o}}{\rm{t}}{\rm{a}}{\rm{l}}\,{\rm{f}}{\rm{l}}{\rm{a}}{\rm{v}}{\rm{o}}{\rm{n}}{\rm{o}}{\rm{i}}{\rm{d}}\,{\rm{u}}{\rm{s}}{\rm{e}}{\rm{d}})\times 100$$

### A. castellanii cultures

*A. castellanii* (ATCC 50492) a clinical strain belonging to the T4 genotype, was routinely cultured in 10 mL growth medium consisting of 0.75% w/v proteose peptone, 0.75% w/v yeast extract, and 1.5% w/v glucose (PYG) in 75-cm^2^ tissue culture flasks at 30 °C as described previously^27^. Amoebicidal and encystation assays were performed with healthy *A. castellanii* trophozoites which are adherent to the surface of tissue culture flask. These active trophozoites were detached by putting culture flasks on ice for 15 min followed by gentle tapping for roughly 5 minutes after changing PYG medium with phosphate buffer saline (PBS) to remove any unhealthy amoeba. Finally, *A. castellanii* trophozoites suspension was centrifuged at 2500 × g for 10 min to obtain amoeba pellet. The pellet was resuspended in 1 mL PBS, and the population of *A. castellanii* was determined by cell counting using a hemocytometer. 5 × 10^5^ *A. castellanii* were used for amoebicidal and encystation assays.

### Henrietta lacks cervical adenocarcinoma (HeLa) cells culture

HeLa cells were cultured in Roswell Park Memorial Institute (RPMI)−1640 supplemented with 10% fetal bovine serum (FBS), 10% Nu-serum, 2 mM glutamine, 1 mM pyruvate, penicillin and streptomycin (100 units/mL and 100 μg/mL respectively), non-essential amino acids, and vitamins to obtain uniform monolayers of cells in 75-cm^2^ culture flasks as described previously^28^. Old media was aspirated, and cells were trypsinized with 2 mL trypsin. The cell suspension was centrifuged for 5 min at 2000 × g, and cell pellet was resuspended in 30 mL fresh cell growth media. 200 μL of this cell suspension was seeded in each well of a 96-well plate and the plate was incubated at 37 °C in a 5% CO_2_ incubator with 95% humidity for at least 24 h until formation of uniform monolayer of HeLa cells. These were used for *N. fowleri* cultures, cytotoxicity, and cytopathogenicity assays.

### N. fowleri cultures

*N. fowleri* (ATCC 30174) a clinical isolate from the cerebrospinal fluid of a patient was cultured in 75-cm^2^ tissue culture flasks containing HeLa monolayers as feed. *N. fowleri* was grown at 37 °C in a 5% CO_2_ incubator with 95% humidity as described previously^21^.

### Amoebicidal assay

The amoebicidal effects of gums, HDN, NRG, gum stabilized nanoparticles, and these nanoparticles loaded with HDN and NRG were tested against *A. castellanii* and *N. fowleri*. 5 × 10^5^ amoebae per well were incubated with nanoparticles and respective controls at 50 and 25 µg per mL in 24-well plates in RPMI-1640 medium at 30 °C for 24 h as reported previously^27^. Next, amoebae viability was determined by Trypan blue (0.1%) exclusion assay. The number of nonstained amoebae (live) were counted using a hemocytometer to estimate the viability. Untreated amoebae were used as negative control, while *A. castellanii* incubated with Chlorhexidine, and *N*. *fowleri* treated with Amphotericin B were considered as positive control. The results are representatives of several experiments presented as the mean ± standard error.

### Encystation assay

Encystation assay was done to test the effects of these nanoparticles to inhibit the differentiation of trophozoites of *A. castellanii* into cysts. 5 × 10^5^ *A. castellanii* were incubated with 100 µg per mL concentrations of gums, HDN, NRG, gum stabilized nanoparticles, and these nanoparticles loaded with HDN and NRG in 1.5 mL centrifuge tubes at room temperature for 10 min. 50 mM MgCl_2_ and 10% glucose was added in 24-well plate as encystation medium, and test samples treated and control *A. castellanii* were added in above 24-well plates. The cells were incubated at 30 °C for 72 h^29^. After 72 h, each well was treated with 0.25% sodium dodecyl sulfate (SDS) to solubilize the trophozoites and only the SDS resistant cysts were tallied using a hemocytometer. The results are representatives of several experiments presented as the mean ± standard error.

### Excystation assay

1 × 10^6^ *A. castellani* trophozoites were harvested on non-nutrient agar plates (1.5% bacteriological agar dissolved in water, followed by autoclaving and then spreading on petri plates). The petri plates were kept in incubator at 30 °C for 14 days, which were routinely observed under light microscope for the formation of cysts. Following this, cysts were scraped using cells scraper with PBS, counted, and stored at 4 °C for further use. 1 × 10^5^ cysts were treated with 100 µg per mL concentrations of HDN and NRG loaded nanoparticles and respective controls in PYG for 72 h^29^. Cysts transformed into trophozoites were enumerated by using hemocytometer. The results are representatives of several experiments presented as the mean ± standard error.

### Bacterial cultures

The bacterial cultures used in this study include neuropathogenic *Escherichia coli* K1 (a cerebrospinal fluid isolate from a meningitis patient; 018:K1:H7), strain E44 {Malaysian Type Culture Collection (MTCC) 710859}, and methicillin-resistant *Staphylococcus aureus* (MRSA) (MTCC 381123). The MRSA strain was originally isolated from blood cultures, obtained from the Luton & Dunstable Hospital NHS Foundation Trust, Luton, England, UK.

### Bactericidal assay

Antibacterial potential of nanoparticles and respective controls was determined by using bactericidal assay as described previously^30^. Briefly, bacterial cultures were fixed to an optical density of 0.22 at 595 nm using a spectrophotometer (OD_595_ = 0.22) which is equivalent to 10^8^ colony-forming units per mL (C.F.U. mL^−1^). An inoculum of 10 μL of above bacteria culture (corresponding approximately 10^6^ C.F.U.) was incubated with various concentrations of GA-AgNPs-HDN, GT-AuNPs-NRG and respective controls in 1.5 mL centrifuge tubes at 37 °C for 2 h. For negative controls untreated bacterial culture were incubated with phosphate buffer saline (PBS), while 100 μg/mL gentamicin treated bacteria were used as positive control. Next, bacteria were serially diluted and 10 µL of each dilution was plated on nutrient agar plates. These plates were incubated at 37 °C overnight, followed by counting viable bacterial C.F.U.

### Pathogens-mediated host cells cytotoxicity

The cytopathogenicity assay was carried out as reported previously^31^. 5 × 10^5^ *A. castellanii*, 10^6^ C.F.U. of each *E. coli* K1 and MRSA were incubated with HDN and NRG loaded nanoparticles with respective controls at different concentrations for 2 h at 30 °C. Next, the microbial cultures were centrifuged at 2500 × g for 10 minutes, and supernatants were wasted to remove extracellular materials. The pellet obtained was resuspended in 500 µL of fresh RPMI-1640 which was put on another 24-well plated with HeLa cells monolayer. Cells were incubated at 37 °C in a 5% CO_2_ incubator with 95% humidity for 24 h. Finally, supernatants were collected from each well and lactate dehydrogenase (LDH) cytotoxicity assay was performed using LDH kit (Roche) as described previously^23^. The extent of LDH release determines cells damage. Untreated cells were considered as negative control, whereas cells incubated with 0.1% Triton X-100 for 20 min gave maximum LDH release as a result of cell lysis which was taken as positive control. The % cell cytotoxicity was calculated as follows: % cell cytotoxicity = (sample absorbance −  negative control absorbance)/(positive control absorbance  −  negative control absorbance) × 100. The results are representatives of several experiments presented as the mean ± standard error.

### Cytotoxicity assay

To evaluate the cytotoxic effects of these nanoparticles on human cell, LDH cytotoxicity assay was performed as reported previously^28^. Briefly, 100 µg per mL concentrations of HDN and NRG loaded nanoparticles and respective controls were treated with uniform monolayer of HeLa cells in a 24-well plate, and the cells were incubated for 24 h at 37 °C in a 5% CO_2_ incubator. After 24 h, supernatants were collected from each well and cytotoxicity was determined by measuring lactate dehydrogenase (LDH) released by using LDH kit (Roche). Untreated cells were considered as negative control, whereas cells incubated with 0.1% Triton X-100 for 20 min gave maximum LDH release as a result of cell lysis which was taken as positive control. The percentage cell cytotoxicity was calculated as follows: % cell cytotoxicity = (sample absorbance − negative control absorbance)/(positive control absorbance − negative control absorbance) × 100. The results are representatives of several experiments presented as the mean ± standard error.

### Statistical analysis

Student T test was used to measure statistical correlation and significance. P < 0.05 was the limit for significance using two-sample T test and two-tailed distribution. *Represents P < 0.05, **represents P < 0.01, while ***represents P < 0.001.

## Results

### Characterization of GA-AgNPs and GA-AgNPs-HDN for determination of size, PDI, zeta potential and surface morphology

**Figure 1:**
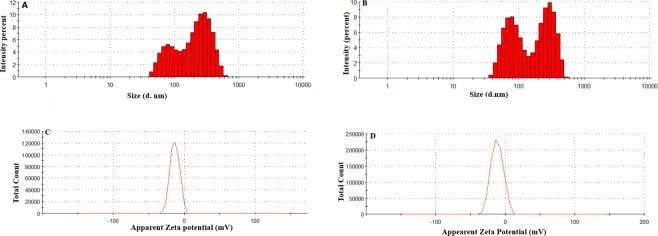
(**A**,**B**) Size distribution histogram of GA-AgNPs and GA-AgNPs-HDN respectively which were obtained from zetasizer analysis on Malvern zetasizer instrument. Mean size distribution of GA-AgNPs and GA-AgNPs-HDN were found to be 107 and 182 nm respectively. The nanoparticles showed larger diameter when loaded with HDN as compared to GA-AgNPs. (**C**,**D**) shows the zeta potential plots for GA-AgNPs and GA-AgNPs-HDN. HDN loading on GA-AgNPs slightly increased the negative surface charge from −18.6 to −19.1 mV which effects the stability.

**Figure 2:**
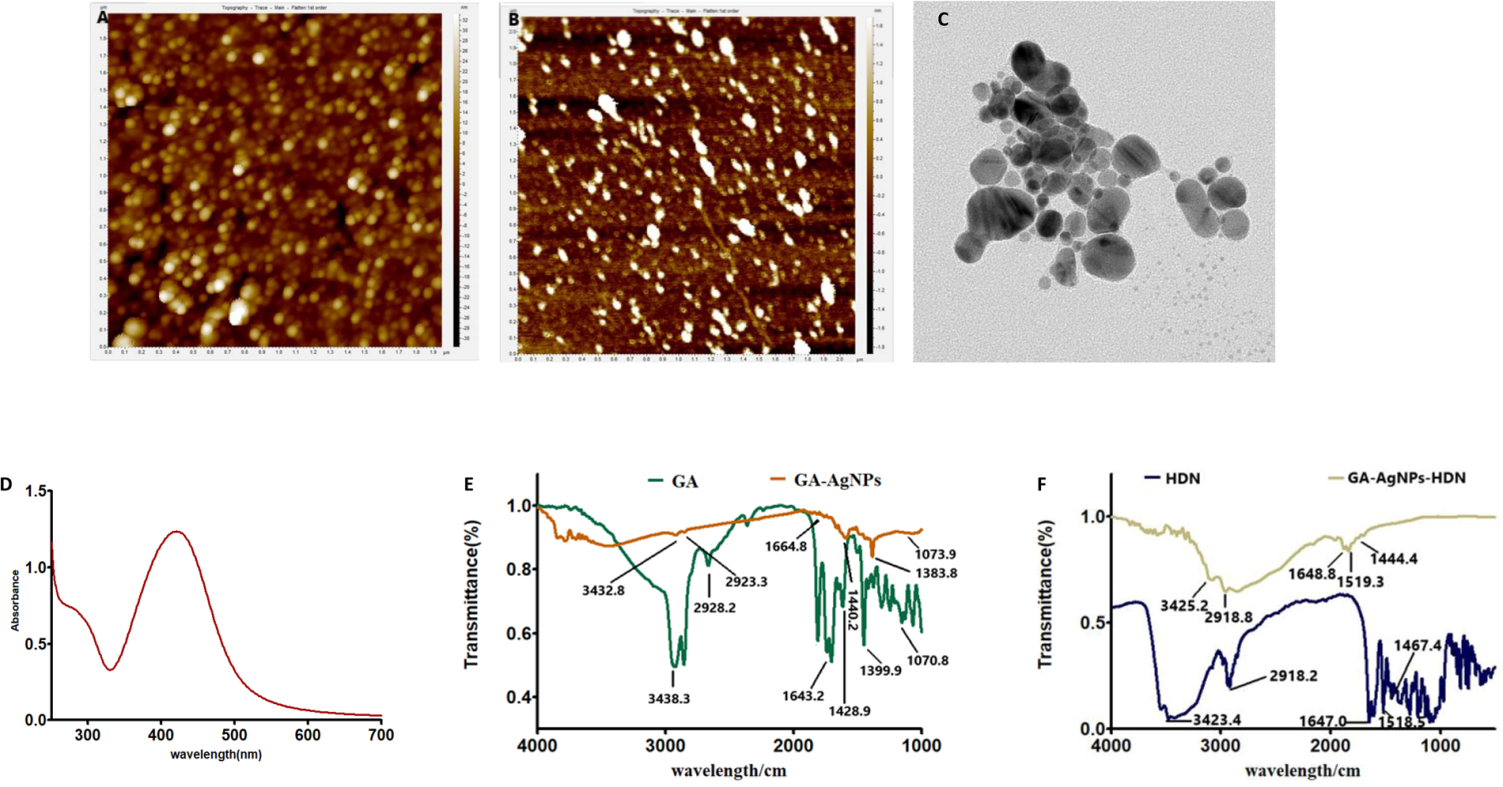
(**A**,**B**) AFM images of GA-AgNPs and GA-AgNPs-HDN respectively, showing formation of spherical nanoparticles. AFM topographic images were recorded at AFM (Agilent 5500) instrument operated in tapping mode with silicon nitride cantilever. (**C**) TEM image of GA-AgNPs-HDN recorded at Tecnai G2 20 S-TWIN instrument. (**D**) UV-vis spectrum of GA-AgNPs-HDN showing characteristic surface plasmon resonance band at 420 nm. UV-visible spectra were recorded at UV-Vis spectrophotometer (Evolution 300, Thermo Scientific) in aqueous medium. (**E**) FT-IR spectra of GA alone is compared with GA-AgNPs. (**F**) Comparative FT-IR analysis of HDN alone and GA-AgNPs-HDN. The spectra were obtained by using FT-IR spectrometer (Shimadzu, Kyoto, Japan).

Size and shape of the nanoparticles have a key importance in the design of drug delivery systems. Smaller the size of the nanoparticles greater will be the surface to volume ratio, therefore the chances of interactions between the bioactive drug molecules and the nanoparticles increases which ultimately enhances the therapeutic efficacy of the drug^32^. GA-AgNPs possess 107.1 ± 2.56 nm mean size with PDI 0.270 ± 0.03. Similarly, GA-AgNPs-HDN exhibit 182.8 ± 1.02 nm and PDI 0.422 ± 0.01 results are shown in Fig. 1A,B. Larger mean size and PDI of the HDN loaded nanoparticles than that of their unloaded analogue is attributed to unequal scattering of the drug moieties over the surface of GA-AgNPs. Zeta potential of GA-AgNPs and GA-AgNPs-HDN was −18.6 ± 0.54 mV and −19.1 ± 1.34 mV respectively as shown in Fig. 1C,D. Zeta potential is another important parameter for the determination of nano-carrier stability. AFM and TEM of GA-Ag-NPs and GA-Ag-NPs-HD were investigated to find their morphology. Both nanoparticles found to be spherical in shape as shown in Fig. 2A–C. A sharp characteristic surface plasmon resonance band of GA-AgNPs-HDN was obtained at 420 nm as shown in Fig. 2D.

### FT-IR analysis of GA-AgNPs and GA-AgNPs-HDN

FT-IR spectrum of GA shows characteristic peaks of a complex mixture of sugars, a stretching band at 3387.8 cm^−1^ assign to N-H, a band at 2931.0 due to asymmetric stretching vibration of C-H groups. Similarly, peak at 1630.7 and 1429.8 cm^−1^ corresponds to amide C=O stretch and C-N stretching vibration. The peak at 1068.1 cm^−1^ is attributed to C-O stretching vibration as shown in Fig. 2E. FT-IR spectrum of GA-AgNPs shows typical peaks at 338.16 and 2924.2 for N-H and C-H respectively. The peak at 1623.4 cm^−1^ corresponds to C=O group, similarly peak at 1431.3 and 1061.7 cm^−1^ due to stretches of C-N and C-O groups of GA. The change in absorbance from 1630.7 to 1623.4 and 1429.8 to 1431.3 and from 1068.1 to 1061.7 cm^−1^ indicates that C=O, C-N and C-O groups are participated in the reduction and stabilization of GA-AgNPs shown in Fig. 2E. The FT-IR spectrum of HDN revealed its distinctive peaks at 3466.1and 2925.50 cm^−1^ assign to OH and C-H groups of HDN. The peak for C=O, C = C and aromatic C=C appeared at 1645.27, 1515.9 and 1443.0 cm^−1^ respectively (Fig. 2F). The FT-IR spectrum of GA-AgNPs-HDN shows all the representative peaks at their respective places with slight changes in absorbance. FT-IR analysis confirms the chemical stability of HDN as all peaks are present on their respective positions in the spectrum shown in Fig. 2F. The drug loading % was found to be 73.66%.

### Characterization of GT-AuNPs and GT-AuNPs-NRG for determination of size, PDI, zeta potential and surface morphology

**Figure 3:**
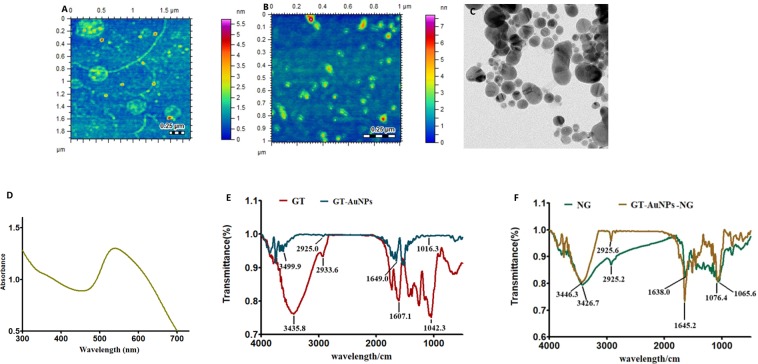
(**A**,**B**) AFM images of GT-AuNPs and GT-AgNPs-NRG respectively, showing formation of spherical nanoparticles. AFM topographic images were recorded at AFM (Agilent 5500) instrument operated in tapping mode with silicon nitride cantilever. (**C**) TEM image of GT-AuNPs-NRG recorded at Tecnai G2 20 S-TWIN instrument. (**D**) UV-vis spectrum of GT-AuNPs-NRG showing characteristic surface plasmon resonance band at 540 nm. UV-visible spectra were recorded at UV-vis spectrophotometer (Evolution 300, Thermo Scientific) in aqueous medium. (**E**) FT-IR spectra of GT alone is compared with GT-AuNPs. (**F**) Comparative FT-IR analysis of NRG alone and GT-AuNPs-NRG. The spectra were obtained by using FT-IR spectrometer (Shimadzu Kyoto, Japan).

GT-AuNPs and GT-AuNPs-NRG exhibit 183 ± 1.04 and 221 ± 1.08 nm mean size with PDI 0.351 ± 0.02 and 0.410 ± 0.03 respectively which are shown in Table 1. The size of the GT-AuNPs as compare to NRG loaded counterpart is relatively larger which confirms the NRG loading on the surface of GT-AuNPs. Zeta potential of GT-AuNPs is −34.1 ± 0.1 and for GT-AuNPs-NRG is −27.6 ± 0.5 mV respectively. The drug loading % was found to be 72%. Surface morphology of both GT-AuNPs and GT-AuNPs-NRG were investigated through AFM and TEM, and they were found to be spherical in shape as shown in Fig. 3A–D. shows a representative surface plasmon resonance band of GT-AuNPs-NRG with maximum absorbance at 540 nm.

**Table 1:** The average size, polydispersity index (PDI), and zeta potential of GT-AuNPs and GT-AuNPs-NRG with the % NRG loading efficiency.

Formulation	Average size (nm)	PDI	Zeta Potential (mV)	% Drug Loading Efficiency
GT-AuNPs	183 ± 1.04	0.351 ± 0.02	−34.1 ± 0.1	-----------
GT-AuNPs-NRG	221 ± 1.08	0.410 ± 0.03	−27.6 ± 0.5	72% ± 1.50

### FT-IR analysis of GT-AuNPs and GT-AuNPs-NRG

FT-IR spectrum of GT shows its distinguishing peaks for -OH and -CH groups at 3435.8 and 2933.6 cm^−1^ respectively. Similarly, typical peaks 1607.1 and 1042.3 cm^−1^ corresponds to -C=O and -C-O groups respectively (Fig. 3E). The FT-IR spectrum of GT-AuNPs shows characteristic vibration of -OH, -CH, stretching of -C=O and stretching of -C–O at 3499.9, 2925.0, 1649.0 and 1016.3 cm^−1^ respectively. The changes in the characteristic peaks of GT from 3435.8 to 3499.9 cm^−1^, 1607.1 to 1649.0 and 1042.3 to 1016.3 cm^−1^ indicate that -OH, -C=O and -C-O functional groups of GT play part in the nanoparticle synthesis. The FT-IR spectrum of NRG shows distinctive peaks at 3426.7 cm for -OH groups and -CH peak at 2925.2 cm^−1^. Similarly, the at 1638.0 and 1065.6 cm^−1^ for -C=O, -C-O groups respectively (Fig. 3F). The FT-IR spectrum of GT-AuNPs-NRG shows predominant shift in peaks from 1065.6 to 1076.4 cm^−1^ and 1638.0 to 1645.2 cm^−1^. This indicates that -C-O and -C=O groups of NRG interacts with GT-AuNPs. The involvement NRG functional groups confirm its loading in GT-AuNPs.

### GA-AgNPs-HDN abolished viability of *A. castellanii* and *N. fowleri*

**Figure 4:**
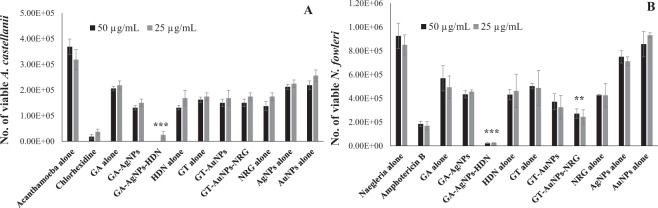
Amoebicidal assay against (**A**) *A. castellanii* and (**B**) *N. fowleri*. The viability of amoebae was determined after amoebicidal assay as described in the materials and methods section. Briefly, *A. castellanii* or *N. fowleri* trophozoites were incubated with GA and GT alone, AgNPs alone, AuNPs alone, HDN alone, NRG alone, GA-AgNPs, GT-AuNPs, GA-AgNPs-HDN, and GT-AuNPs-NRG and negative and positive controls at 50 and 25 µg per mL at 30 °C for 24 h. Next, the viability was measured by Trypan blue exclusion assay. The results are presented as the mean ± standard error of various experiments performed in duplicate. *Represents P < 0.05, **represents P < 0.01, while ***represents P < 0.001. P values were obtained using two-sample T test and two-tailed distribution.

Amoebicidal assay revealed that GA-AgNPs-HDN killed all the *A. castellanii* trophozoites at 50 µg per mL, and significantly reduced the number of cells by 90% at 25 µg per mL as compared to GA alone, HDN alone, and GA-AgNPs (Fig. 4A). Notably GA-AgNPs-HDN was found to be more effective than positive control Chlorhexidine. Whereas, GT-AuNPs-NRG did not produce cidal effects when statistically compared with GT alone, NRG alone and GT-AuNPs. On the other hand, both GA-AgNPs-HDN and GT-AuNPs-NRG exhibited significant amoebicidal effects against *N. fowleri* as compared to gums alone, drugs alone and gums stabilized nanoparticles (Fig. 4B). GA-AgNPs-HDN caused 99% reduction in *N. fowleri* viability at 25 µg per mL which is also significantly more effective than Amphotericin B alone. These results suggest that GA-AgNPs-HDN is an exceptional formulation which hold potential for further studies.

### GA-AgNPs-HDN and GT-AuNPs-NRG inhibited encystment and excystation of *A. castellanii*

**Figure 5:**
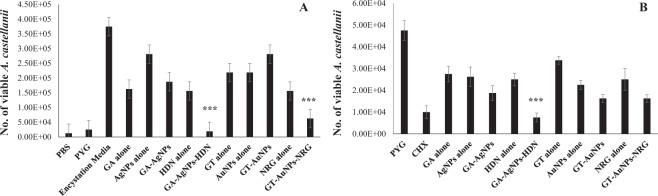
Depiction of the results of (**A**) encystation. GA-AgNPs-HDN inhibited *A. castellanii* encystation. *A. castellanii* (1 × 10^5^) were inoculated in PBS in the presence of GA-AgNPs-HDN and GT-AuNPs-NRG and respective controls at 100 µg per mL with encystation media and incubated at 30 °C for 72 h. Next, 0.25% sodium dodecyl sulfate (SDS) was added and incubated at room temperature for 10 min to lyse *A. castellanii* trophozoites followed by enumeration of amoebae cysts using a hemocytometer. (**B**) Excystation assays was performed by incubating GA-AgNPs, GT-AuNPs, GA-AgNPs-HDN, GT-AuNPs-NRG and respective controls (100 µg per mL) with *A. castellanii* cysts (1 × 10^5^) in growth medium, PYG at 30 °C for 72 h. After this period, amoebae were counted using a hemocytometer. The results are presented as the mean ± standard error of various experiments performed in duplicate. *Represents P < 0.05, **represents P < 0.01, while ***represents P < 0.001. P values were obtained using two-sample T test and two-tailed distribution.

As encystment of *A. castellanii* is responsible for the resistance against drugs, these nanoparticles were tested for inhibition of encystation. GA-AgNPs-HDN and GT-AuNPs-NRG significantly inhibited the encystation of *A. castellanii* at 100 µg per mL as compared to respective controls (Fig. 5A). GA-AgNPs-HDN caused 95% inhibition and GT-AuNPs-NRG inhibited the encystation by 85%. The de-differentiation of cysts into trophozoites causes recurrence of infection in most of the cases. Therefore, the effects of GA-AgNPs-HDN and GT-AuNPs-NRG were also evaluated against excystation. While treated with pre-formed mature cysts of *A. castellanii*, GA-AgNPs-HDN inhibited excystation by 84% at 100 µg per mL (Fig. 5B). Contrary, GT-AuNPs-NRG did not exhibit significant excystation when compared with GT-AuNPs. Since most of the lead compounds and drugs have limited effects against cysts of *A. castellanii*, these nanoparticles showed consistent effects against trophozoite as well as resistant cyst stage.

### GA-AgNPs-HDN exhibited significant bactericidal effects

**Figure 6:**
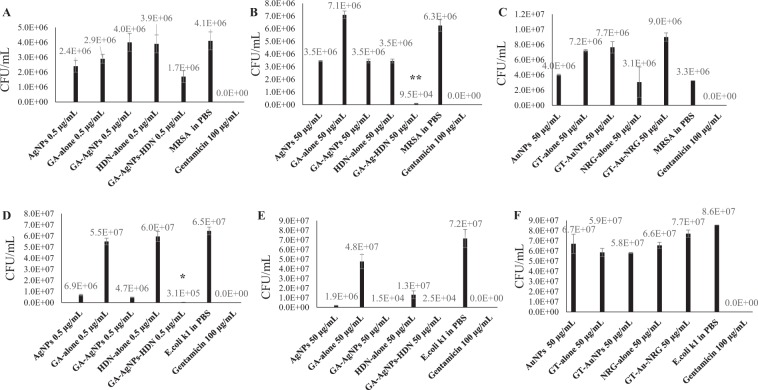
Antibacterial activities of green synthesized nanoparticles against multi-drug resistant bacteria. (**A**–**C**) Bactericidal effects against MRSA. GA-AgNPs-HDN at 0.5 µg per mL **(A)**, and 50 µg per mL (**B**) GT-AuNPs-NRG shows no antibacterial activity (**C**). (**D–F**) Bactericidal effects against *E. coli* K1. GA-AgNPs-HDN at 0.5 µg per mL (**D**), and 50 µg per mL (**E**). GT-AuNPs-NRG shows no antibacterial activity (**F**). The results are presented as the mean ± standard error of various experiments performed in duplicate. *Represents P < 0.05, **represents P < 0.01, while ***represents P < 0.001. P values were obtained using two-sample T test and two-tailed distribution.

**Figure 7:**
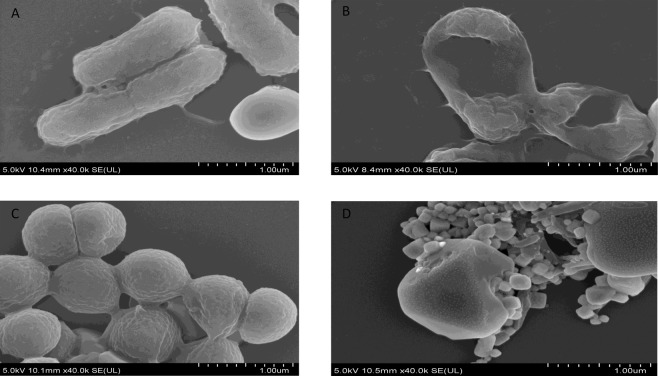
FE-SEM images of bacteria treated with GA-AgNPs-HDN. Bacteria treated with nanoparticles were fixed on glass cover slips by using glutaraldehyde. Followed by fixation, the samples were washed with ethanol and images were recorded on Field-emission scanning electron microscope (FE-SEM) (Hitachi SU8010) instrument. (**A**) *E. coli* K1 control. (**B**) *E. coli* K1 treated with 0.5 µg per mL of GA-AgNPs-HDN. (**C**) MRSA control. (**D**) MRSA treated with 50 µg per mL of GA-AgNPs-HDN. Control bacteria show integrated cell membrane, whereas treated bacteria show disintegration and pores.

Figure 6 represents the bactericidal effects of GA-AgNPs-HDN and GT-AuNPs-NRG tested at 50 and 0.5 µg per mL against MRSA and *E. coli* K1. GA-AgNPs-HDN showed significant bactericidal activity at 50 µg per mL against MRSA (Fig. 6B), and 0.5 µg per mL against *E. coli* K1 (Fig. 6D). GT-AuNPs-NRG did not exhibit bactericidal effects at 50 µg per mL against both tested bacteria (Fig. 6C,F). Figure 7 presents the corresponding field emission scanning electron microscopic (FE-SEM) analysis of bacteria before and after treatment with GA-AgNPs-HDN.

### GA-AgNPs-HDN reduced the pathogens-mediated host cell cytotoxicity

**Figure 8:**
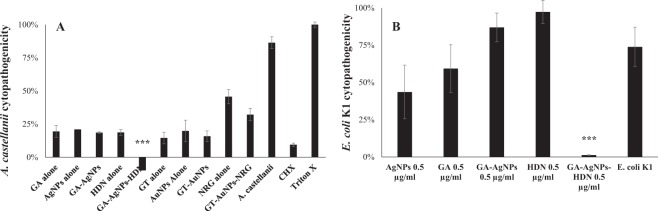
(**A**) Pretreatment of 100 µg per mL of GA-AgNPs-HDN abolished *A. castellanii*-mediated host cells cytotoxicity. Briefly, amoebae (1 × 10^5^) were incubated at 30 °C with GA-AgNPs-HDN, GT-AuNPs-NRG and respective controls for 2 h in RPMI-1640 and then incubated with HeLa cells for 24 h at 37 °C in a 5% CO_2_ incubator as described in materials and methods section. Next, cell-free supernatant was collected, and cytotoxicity was determined using Lactate dehydrogenase (LDH) assay kit (Roche). (**B**) *E. coli* K1 caused 74% cytotoxicity to HeLa cells. Upon pretreatment with 0.5 µg per mL GA-AgNPs-HDN, the host cells cytotoxicity was reduced to 1%. The results are presented as the mean ± standard error of various experiments performed in duplicate. *Represents P < 0.05, **represents P < 0.01, while ***represents P < 0.001. P values were obtained using two-sample T test and two-tailed distribution.

The pretreatment of *A. castellanii* and *E. coli* K1 with GA-AgNPs-HDN resulted in significant reduction of their cytopathogenicity against human cells. Figure 8A describes that untreated *A. castellanii* caused more than 80% cell cytotoxicity against HeLa cells, contrary, GA-AgNPs-HDN (50 µg per mL) completely obliterated the host cells cytotoxicity as compared to relative controls. Similarly, the pretreatment of 0.5 µg per mL GA-AgNPs-HDN with *E. coli* K1 abolished the cytotoxicity of bacterium against HeLa cells as compared to untreated *E. coli* K1 which exhibited 74% cytotoxicity (Fig. 8B).

### GA-AgNPs-HDN and GT-AuNPs-NRG showed minimal cytotoxicity against human cells

**Figure 9:**
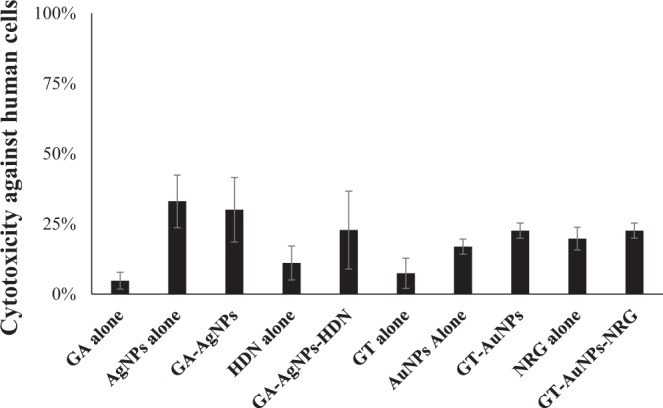
GA-AgNPs-HDN and GT-AuNPs-NRG did not exhibit cytotoxicity against HeLa cells at 100 µg per mL. These nanoparticles and the respective controls were incubated at 30 °C with HeLa cells monolayer for 24 h at 37 °C in a 5% CO_2_ incubator. Following this incubation, cell-free supernatant was collected, and cytotoxicity was determined using Lactate dehydrogenase (LDH) assay kit (Roche). The negative control values for cytotoxicity assays were obtained by incubating HeLa cells with RPMI-1640 alone, and positive control values were obtained by 100% cell death using 0.1% Triton X-100. The results are presented as the mean ± standard error of various experiments performed in duplicate.

When tested against human cells, all test samples showed minimal cytotoxic effects (Fig. 9). GA-AgNPs-HDN exhibited only 11% cytotoxicity while GT-AuNPs-NRG caused 23% cytotoxicity against HeLa at a higher concentration of 100 µg per mL as compared to amoebicidal effects which were recorded at 50 and 25 µg per mL. The cytotoxicity profile against human cells suggest that these nanoparticles are biosafe and can further be evaluated for potential in *in vivo* studies.

## Discussion

Brain-eating amoebae are opportunistic protist pathogens associated with diseases of fatal severity. The molecular pathways to target these microbes are limited which results in challenges in development of effective therapeutics^4^. Current management and treatment are unspecific and ineffective due to which the CNS infections caused by brain-eating amoebae almost always proved to be deadly^33^. Furthermore, the clinical procedures suffer from limitations including long term use of medications (a mixture of drugs including biguanides, azoles, amidines, antibiotics) and still the chances of recurrence are high^34^. On the other hand, ever growing drug resistance in most commonly present bacteria, and lack of newer and improved antimicrobial agents pose serious challenges to healthcare systems^35^. Therefore, there is an urgent need to develop novel, sustainable, and effective modalities of chemotherapeutics against infectious diseases. Nanotechnology has proved to be a model alternative to target infectious diseases^36^. Due to small size of nanomaterials, these are efficient drug delivery carriers for minimizing the pharmacokinetics and pharmacodynamics limitations of compounds and drugs known to have medicinal values^37^. Flavonoids are important nutraceutical and biologically active class of secondary metabolites natural products. Flavonoids obtained from citrus fruit plants are rich of drug candidates against a variety of diseases such as; infectious diseases, cancer, neurodegenerative etc.^38–41^. However, their clinical applications have some common shortcomings, and their poor bioavailability is one of the major factors^14^. In this study, we synthesized silver and gold nanoparticles stabilized with plant gums and loaded them with two most common citrus fruits flavonoids HDN and NRG to utilize their antimicrobial activity against brain-eating parasites *A. castellanii* and *N. fowleri* and multi-drug resistant bacteria MRSA and neuropathogenic *E. coli* K1.

Green synthesis of nanoparticles involves the reduction of metal ions by using environmentally and eco-friendly materials which act as reducing and stabilizing agents. Microorganisms and plant materials have been widely used for the biosynthesis of nanoparticles^42^. Green synthesized nanoparticles have been extensively used against microbial diseases, however, only few results are reported against parasitic diseases^43^. Besides metallic nanoparticles, green polymers including cellulose and starch have also been used for clinical and biomedical applications including bones healing and substitution^44–46^. The synthesis and stabilization of nanoparticles is dependent on the reducing and capping ability of the material used. In this study, the reduction of silver and gold is accomplished by using biocompatible natural gums; GA and GT. This green approach is being exploited for the formation of nanoparticles by avoiding any toxic reducing agent and harsh temperature conditions^17^. These nanoparticles were thoroughly characterized by various instrumental techniques before subjected to biological evaluation against brain-eating amoebae.

The role of Toll-like receptors (TLRs) in innate immune responses to pathogens is well recognized^47,48^. Our previous study showed that, HDN is known to reduce the expression of mRNA in TLRs which as a result reduce inflammation^17^. As the TLRs can influence the immunopathogenesis of CNS parasitic infections, we proposed that using TLRs targeting compounds can decrease the activity of inflammatory cytokines which may affect the parasite clearance and host survival. However, upon loading of HDN on GA-AgNPs caused surprisingly drastic amoebicidal effects, the mechanism of which is yet unknown. On the other hand, NRG acts as inhibitor of cytochrome P450^49^, which is known to be a common pathway associated with antimicrobial mode of action against brain-eating amoebae^50^. In our previous report, we showed the antibacterial effects of GT-AuNPs-NRG against a variety of bacteria, however their IC_50_ values were high (in the range of 250–300 µg per mL)^26^. GA-AgNPs-HDN are found to be more potent which showed significant bactericidal effects at 50 and 0.5 µg per mL against MRSA and *E. coli* K1 respectively. Interestingly, Gram-negative *E. coli* K1 which have additional peptidoglycan cell wall as compared to Gram-positive bacteria is found to be more susceptible to GA-AgNPs-HDN. The mode of action of such potent antimicrobial effects of these nanoparticles however is still to be determined.

## Conclusions

The green synthesis of silver and gold nanoparticles stabilized with natural glycosidic polymers of plant gums (gum acacia and gum tragacanth) was achieved. These nanoparticles were further loaded with citrus fruits flavonoids HDN and NRG to obtain GA-AgNPs-HDN and GT-AuNPs-NRG. The nanoparticles were characterized by zetasizer, zetapotential, AFM, UV-vis spectrophotometric, and FT-IR analyses. GA-AgNPs-HDN and GT-AuNPs-NRG were tested against brain-eating amoebae *A. castellanii* and *N. fowleri*, as well as multi-drug resistant bacteria MRSA and neuropathogenic *E. coli* K1. These nanoparticles exhibited potent amoebicidal and bactericidal effects, and also inhibited the encystation and excystation processes of *A. castellanii*. Furthermore, these nanoparticles significantly reduced the pathogens-mediated host cells cytotoxicity. Interestingly, these nanocarriers did not show cytotoxicity against human cells even at higher concentration as compared to their concentration used for antimicrobial effects. This study demonstrates a potential development of effective antimicrobial nano-formulations based on naturally occurring flavonoids. These results are anticipated to be a major step forward in developing efficient nanomedicine against pathogenic microbes including brain-eating amoebae and bacterial infections. The mechanism of action and *in vivo* studies are part of our future research.

## Data Availability

Data will be provided upon request on case to case basis.
